# CircRNA hsa_circ_0006215 promotes osteogenic differentiation of BMSCs and enhances osteogenesis–angiogenesis coupling by competitively binding to miR-942-5p and regulating RUNX2 and VEGF

**DOI:** 10.18632/aging.202791

**Published:** 2021-04-04

**Authors:** Houlin Ji, Xu Cui, Yang Yang, Xiaoxiao Zhou

**Affiliations:** 1 Graduate School of Shanghai University of Traditional Chinese Medicine, Shanghai, China; 2 Department of Orthopedics, Shanghai University of Medicine and Health Sciences Affiliated Zhoupu Hospital, Shanghai, China; 3 Department of Orthopedics, Taizhou Hospital of Zhejiang Province, Zhejiang, China; 4 Shanghai University of Traditional Chinese Medicine, Shanghai, China

**Keywords:** senile osteoporosis, circular RNA, bone marrow mesenchymal stem cells, angiogenesis, osteogenesis

## Abstract

Coupling between osteogenesis and angiogenesis determines bone morphology. A decrease in the osteogenic ability of bone marrow mesenchymal stem cells (BMSCs) is one of the underlying causes of senile osteoporosis (OP). Here, we investigated the involvement of circular RNAs (circRNAs) in the osteogenic differentiation of BMSCs and the pathogenesis of senile OP. We sequenced RNA and found decreases expression of hsa_circ_0006215 in BMSCs from patients with OP. We further assessed the role of hsa_circ_0006215 in the osteogenic differentiation of BMSCs using lentivirus-mediated hsa_circ_0006215 overexpression and knockdown. Overexpression of hsa_circ_0006215 promoted the osteogenic differentiation of BMSCs. Luciferase reporter and RNA pull-down assays revealed that hsa_circ_0006215 bound to miRNA-942-5p and thus regulated RUNX2 and vascular endothelial growth factor (VEGF) expression in BMSCs. We assessed osteogenesis and vascular coupling in co-cultured cells, and the role of hsa_circ_0006215 in bone formation *in vivo* using a cortical bone defect model. We found that hsa_circ_0006215 promoted bone defect repair. Overall, our results showed that hsa_circ_0006215 has an important function in osteogenesis and could be a novel target for treating senile OP.

## INTRODUCTION

The National Institutes of Health (2001) proposed that age-related senile osteoporosis (OP) is a disease of the skeletal system characterized by decreased bone strength and increased fracture risk. Its severity is second only to that of cardiovascular diseases and its incidence is linearly associated with the global increase in elderly populations. Osteoporosis now seriously endangers the health of elderly persons [1]. The prevalence of OP in the elderly population of China is ~ 36%. Osteoporotic fracture due to low bone mass, bone microstructure damage, and increased bone fragility is one of the most common and serious complications of senile OP. Every hour, 1,000 patients are being diagnosed with osteoporotic fractures across the world, and the disability and mortality rates are 50% and 42%, respectively.

Bone mineral density (BMD) and quality are reflected in bone strength. Bone formation involves the proliferation and maturation of osteoblasts, and mineralization of the extracellular matrix. Bone is mainly surrounded by matrix and fibers secreted by osteoblasts, which constitute osteoid [2]. Bone tissue is formed after calcium salts are deposited in the osteoid, thus osteoblasts are the main cell type involved in bone formation. They are derived from bone marrow mesenchymal stem cells (BMSCs) and are regulated *in vivo* by several factors. The mechanisms underlying BMSCs differentiation into osteoblasts are obscure. However, many self-regulatory (e.g., transcription factors that control the expression of genes involved in this process) and environmental (e.g., secretory and chemical factors, extracellular matrix, and cell–cell interaction) factors influence BMSCs differentiation [3–5]. Coupling between bone formation and angiogenesis determines bone morphology. Bone homeostasis is destroyed when the bone formation and angiogenesis decrease [6–8]. This happens gradually with advancing age and results in senile OP. In individuals with senile OP, hip and spine fractures can occur due to mild trauma, or even without trauma, and can lead to pain, dysfunction, and even death. These complications seriously affect the health and quality of life of the elderly, and can shorten their life span. Therefore, the early diagnosis, prevention, and interventional treatment of senile OP are important [9, 10].

In bone marrow, BMSCs besides being hematopoietic stem cells, also have potential for self-renewal and multidirectional differentiation [2, 11]. A reduction in osteogenesis is closely associated with the direction of BMSCs differentiation. A decreased differentiation of BMSCs into osteoblasts and increased adipogenic differentiation are important mechanisms underlying the pathogenesis of senile OP. Therefore, identifying key factors to determine the direction of BMSCs differentiation may provide new ideas for the research and treatment of senile OP.

Circular RNAs (circRNAs) are at the global frontiers of life science research [12]. They comprise a class of non-coding RNAs (ncRNAs) that differ from linear RNA as they do not have a 5′-end cap or a 3′-end poly (A) tail and form ring structures with covalent bonds. They are produced mostly from exons and are found in almost all organisms. Circular RNAs were discovered in RNA viruses with ~1500 nucleotides (nt). The closed ring structure of circRNAs render them resistant to nucleic acid exonuclease (RNase) degradation and more stable than linear RNA [13]. Circular RNA are spatial, temporal, and disease specific [14], are expressed in various cells and tissues, and can potentially serve as biomarkers and therapeutic targets. The expression profiles of circRNAs are specific in many pathologic states, including Alzheimer disease, diabetes mellitus, and malignant tumors [15]. Although circRNAs are mainly located in the cytoplasm, a few containing introns originate in the nucleus. The average length of circRNAs are 547 nucleotides, with a range between hundreds and thousands, and the half-life of most circRNAs are > 48 h. Importantly, circRNAs are generated by non-standard variable splicing, which connects splicing donors with upstream splicing acceptors and forms covalently closed rings. The role of circRNAs in the differentiation of BMSCs into osteoblasts remains unclear. The present study thus, aimed to determine how circRNA affect the osteogenic differentiation of BMSCs *in vitro* and in *vivo*.

## RESULTS

### CircRNA hsa_circ_0006215 is downregulated in BMSCs from patients with OP

**Figure 1:**
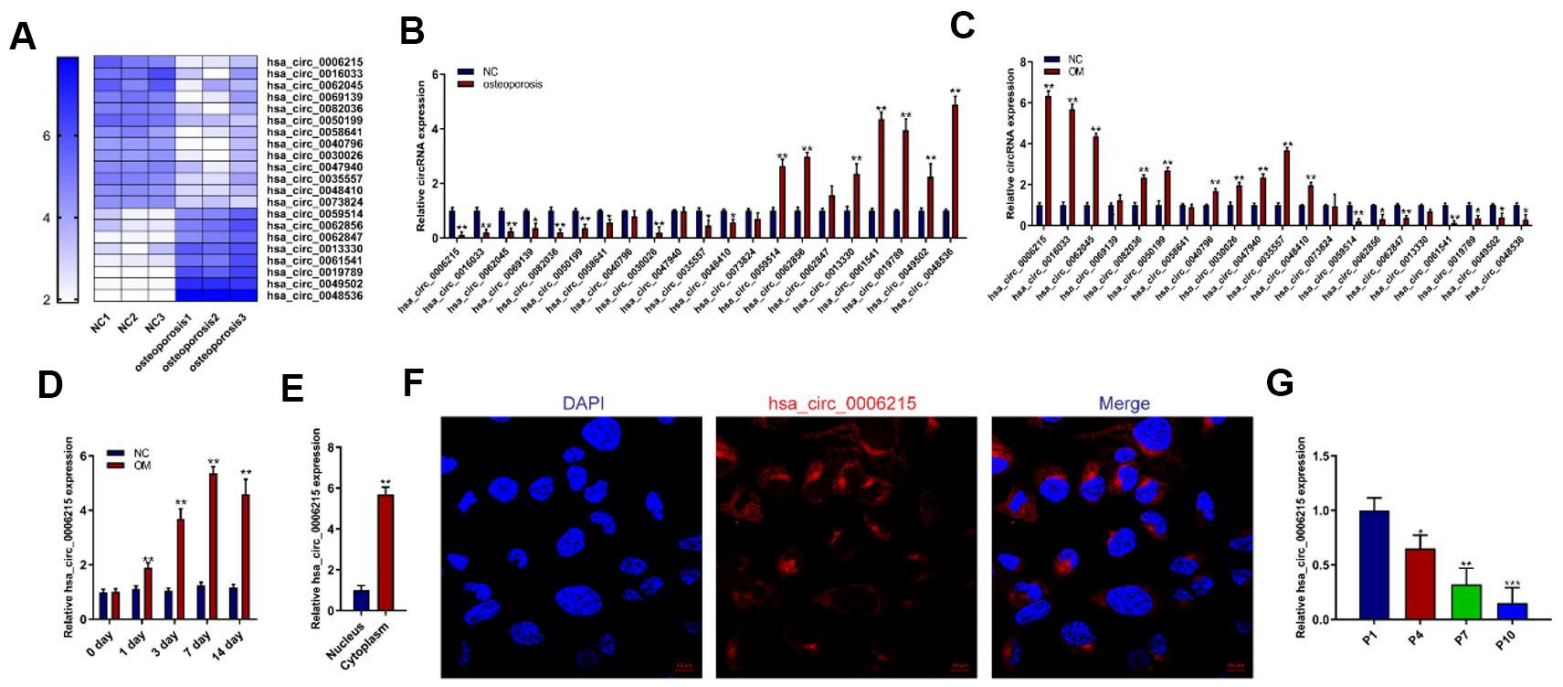
**CircRNA hsa_circ_0006215 downregulates in BMSCs isolated from patients with osteoporosis.** (**A**) Heat map of circRNA RNA-seq. Expression of circRNAs using qRT-PCR in (**B**) BMSCs from patients with and without senile osteoporosis and (**C**) BMSCs during osteogenic differentiation. Expression of hsa_circ_0006215 detected by qRT-PCR (**D**) on days 0, 1, 3, 7 and 14 of osteogenic differentiation and (**E**) in nucleus and cytoplasm. (**F**) Distribution of hsa_circ_0006215 in BMSCs detected by FISH. (**G**) Expression of hsa_circ_0006215 in BMSCs at different passages detected by qRT-PCR. Results are expressed as means ± SD of three independent experiments. *p < 0.05, **p < 0.01. FISH, fluorescent *in situ* hybridization.

We used RNA-seq to assess the expression of circRNAs in BMSCs collected from individuals with or without OP to determine their role in senile OP. The expression of circRNA in BMSCs considerably differed between the two groups (Figure 1A). The RNA-seq results were validated by quantitative real-time polymerase chain reaction (qRT-PCR). Expression of the circRNA hsa_circ_0006215 in BMSCs from patients with OP was significantly decreased compared with the normal controls (Figure 1B). We also determined circRNA that were differentially expressed in BMSCs during osteogenic differentiation. The expression of hsa_circ_0006215 significantly increased during the osteogenic differentiation of BMSCs and peaked on the 7^th^ day of induced osteogenic differentiation (Figure 1C, 1D). Quantitative RT-PCR using total RNA extracted from the nucleus and cytoplasm showed that hsa_circ_0006215 mainly localized in the cytoplasm (Figure 1E), which was also confirmed by results of fluorescence *in situ* hybridization (Figure 1F). An important cause of senile OP is aging BMSCs. Therefore, we constructed a replicative model of aging BMSCs *in vitro*, and found that hsa_circ_0006215 expression significantly decreased in BMSCs as the number of replications increased (Figure 1G).

### CircRNA hsa_circ_0006215 promotes BMSCs osteogenesis

**Figure 2:**
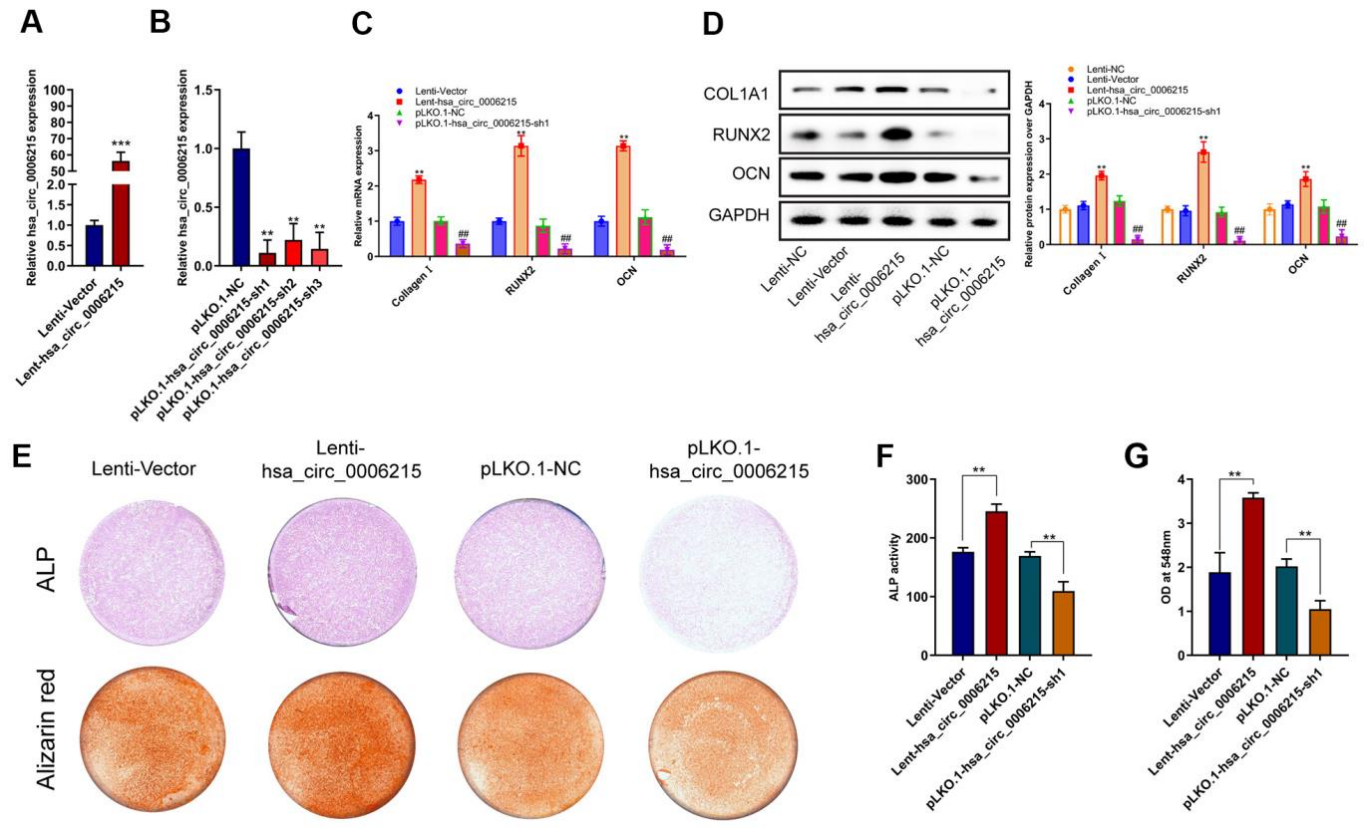
**CircRNA hsa_circ_0006215 promote BMSCs osteogenesis.** Overexpression (**A**), and expression of knocked down (**B**) hsa_circ_0006215 in BMSCs determined by qRT-PCR. Expression of COL1A1, RUNX2 and OCN in BMSCs after overexpression and knockdown of hsa_circ_0006215 detected by qRT-PCR (**C**) and western blotting (**D**). Osteogenic differentiation of BMSCs after overexpression and knockdown of hsa_circ_0006215 detected by ALP and alizarin red staining (**E**). (**F**) Quantitative analysis of osteogenic differentiation of BMSCs after overexpression and knockdown of hsa_circ_0006215 using ALP (**F**, **G**). All values are expressed as means ± SD of three independent experiments. *p < 0.05, **p < 0.01.

We further investigated the mechanism underlying the role of hsa_circ_0006215 in the osteogenic differentiation of BMSCs using lentiviruses to overexpress and knockdown hsa_circ_0006215 in BMSCs. The qRT-PCR results showed that hsa_circ_0006215 overexpression and knockdown significantly increased and decreased the expression of hsa_circ_0006215, respectively, in BMSCs (Figure 2A, 2B). The overexpression of hsa_circ_0006215 also enhanced expression of the osteogenesis-related genes, *COL1A1*, *RUNX2*, and *OCN* (Figure 2C). In contrast, the expression of these genes was decreased when hsa_circ_0006215 was knocked down (Figure 2C). Results of western blotting revealed similar findings at the protein level (Figure 2D). The quantitative findings of alkaline phosphatase (ALP) and alizarin red staining showed that hsa_circ_0006215 overexpression and knockdown significantly promoted and inhibited the osteogenic differentiation of BMSCs, respectively (Figure 2E–2G).

### CircRNA hsa_circ_0006215 is a ceRNA for miR-942-5p and regulates RUNX2 and VEGF expression

**Figure 3:**
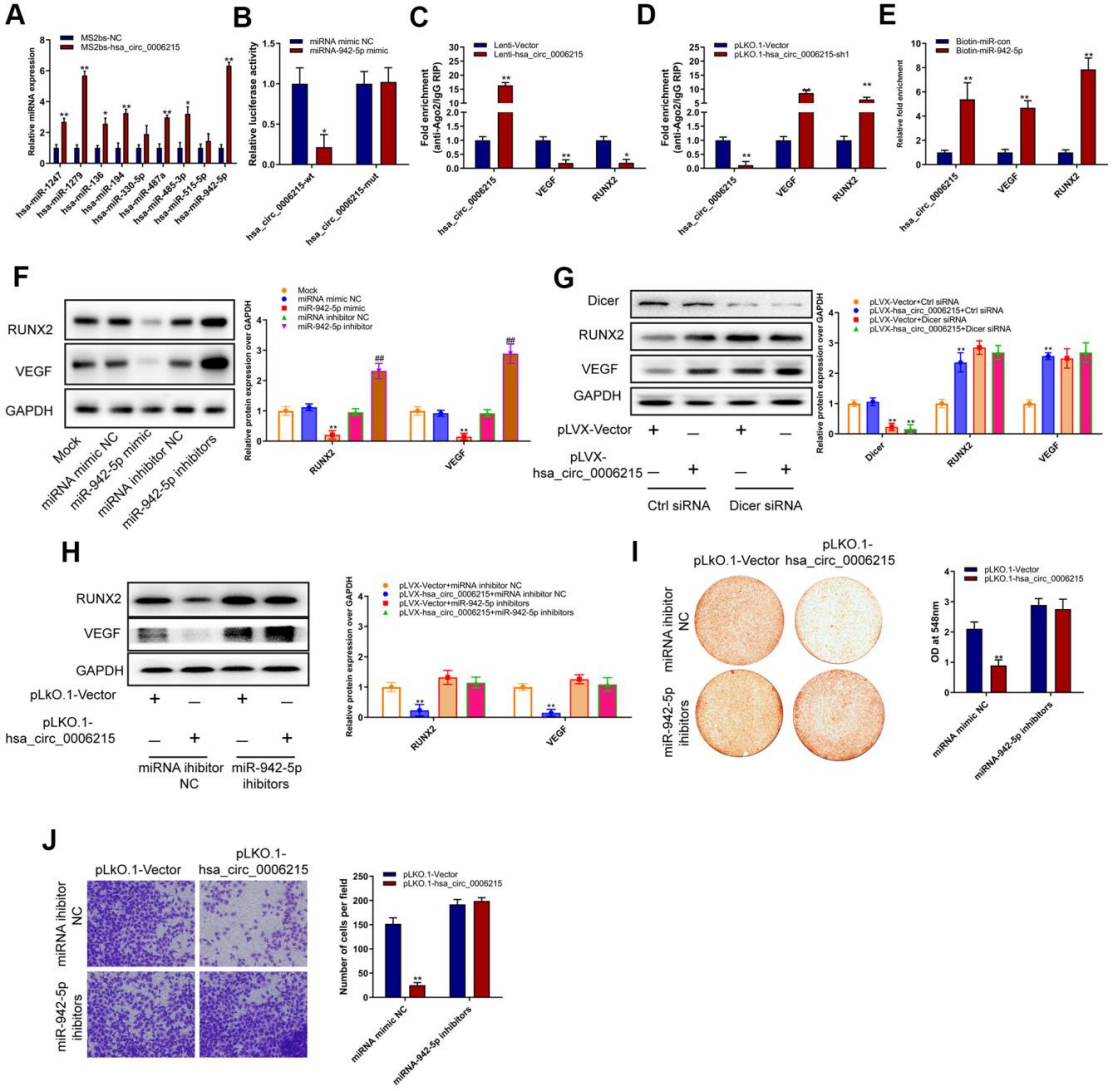
**CircRNA hsa_circ_0006215 functions as a ceRNA for miR-942-5p regulate the expression of RUNX2 and VEGF.** (**A**) Enrichment of microRNAs by hsa_circ_0006215. (**B**) Binding of hsa_circ_0006215 to microRNA-942-5p detected using Luciferase reporter gene. (**C**, **D**) Binding of hsa_circ_0006215, VEGF and RUNX2 to Ago2 determined by Ago2 RIP. (**E**) Binding of microRNA to hsa_circ_0006215 determined by microRNA pull down. Western blots show effects of (**F**) miRNA-942-5p, miRNA-942-5p mimics or inhibitors (CACAUGGCCGAAACAGAGAAG) on RUNX2 and VEGF protein expression, (**G**) hsa_circ_0006215 on RUNX2 and VEGF protein expression, (**H**) hsa_circ_0006215 on of RUNX2 and VEGF protein expression after inhibiting microRNA-942-5p function. Effects of hsa_circ_0006215 on (**I**) BMSC osteogenesis after inhibiting microRNA-942-5p function detected by Alizarin red staining, (**J**) invasion of HUVEC after inhibiting microRNA-942-5p function using Transwell™ assays. Results are expressed as means ± SD of three independent experiments. *p < 0.05, **p < 0.01.

Based on our finding that hsa_circ_0006215 mainly localized in the cytoplasm, we speculated that it regulates miRNA expression via the competitive endogenous RNA (ceRNA) mechanism. We used online bioinformatics software (Circular RNA Interactome https://circinteractome.nia.nih.gov/divergent_primers.html) and found that hsa_circ_0006215 has binding sites for several miRNA. We validated this using RNA pull-down assays, in which we enriched several miRNAs by pulling them down with linearized circRNA hsa_circ_0006215; miRNAs-942-5p was the most enriched among all the miRNAs (Figure 3A). Mutations of the gene encoding hsa_circ_0006215 in the region involved in binding miRNA-942-5p did not affect luciferase activity in luciferase reporter assays; however, miRNA-942-5p significantly affected the luciferase activity of the vector carrying the wild-type hsa_circ_0006215 gene (Figure 3B). The results of TargetScan analysis showed that microRNA-942-5p bind to the RUNX2 and VEGF 3′-UTR. Ago2 RNA immunoprecipitation (RIP) assays showed that overexpressed hsa_circ_0006215 significantly inhibited the binding of Ago2 and VEGF and RUNX2, whereas inhibiting hsa_circ_0006215 expression increased that of Ago2, VEGF, and RUNX2 (Figure 3C, 3D). These results showed that miR-942-5p binds to hsa_circ_0006215, VEGF, and RUNX2 (Figure 3E). Overexpressed hsa_circ_0006215 indirectly promoted RUNX2 and VEGF protein expression by binding more miRNA-942-5p. Knocked down Dicer expression was accompanied by decreased expression of miRNA-942-5p, and reduced promotional effect of hsa_circ_0006215 on the expression of RUNX2 and VEGF, indicating miR-942-5p dependence (Figure 3G). Inhibition of hsa_circ_0006215 expression decreased expression of RUNX2 and VEGF, and inhibiting miRNA-942-5p function decreased the regulatory effects of hsa_circ_0006215 on the expression of RUNX2 and VEGF (Figure 3H). Inhibiting hsa_circ_0006215 expression decreased BMSCs osteogenesis, whereas inhibiting miRNA-942-5p minimized this decrease (Figure 3I). Inhibiting hsa_circ_0006215 expression decreased the invasion of human umbilical vein endothelial cells (HUVECs; American Type Culture Collection, Manassas, VA, USA), while inhibiting miRNA-942-5p decreased the regulatory effects of hsa_circ_0006215 on invasion of HUVECs (Figure 3J).

### CircRNA hsa_circ_0006215 promotes the osteogenesis–angiogenesis coupling

**Figure 4:**
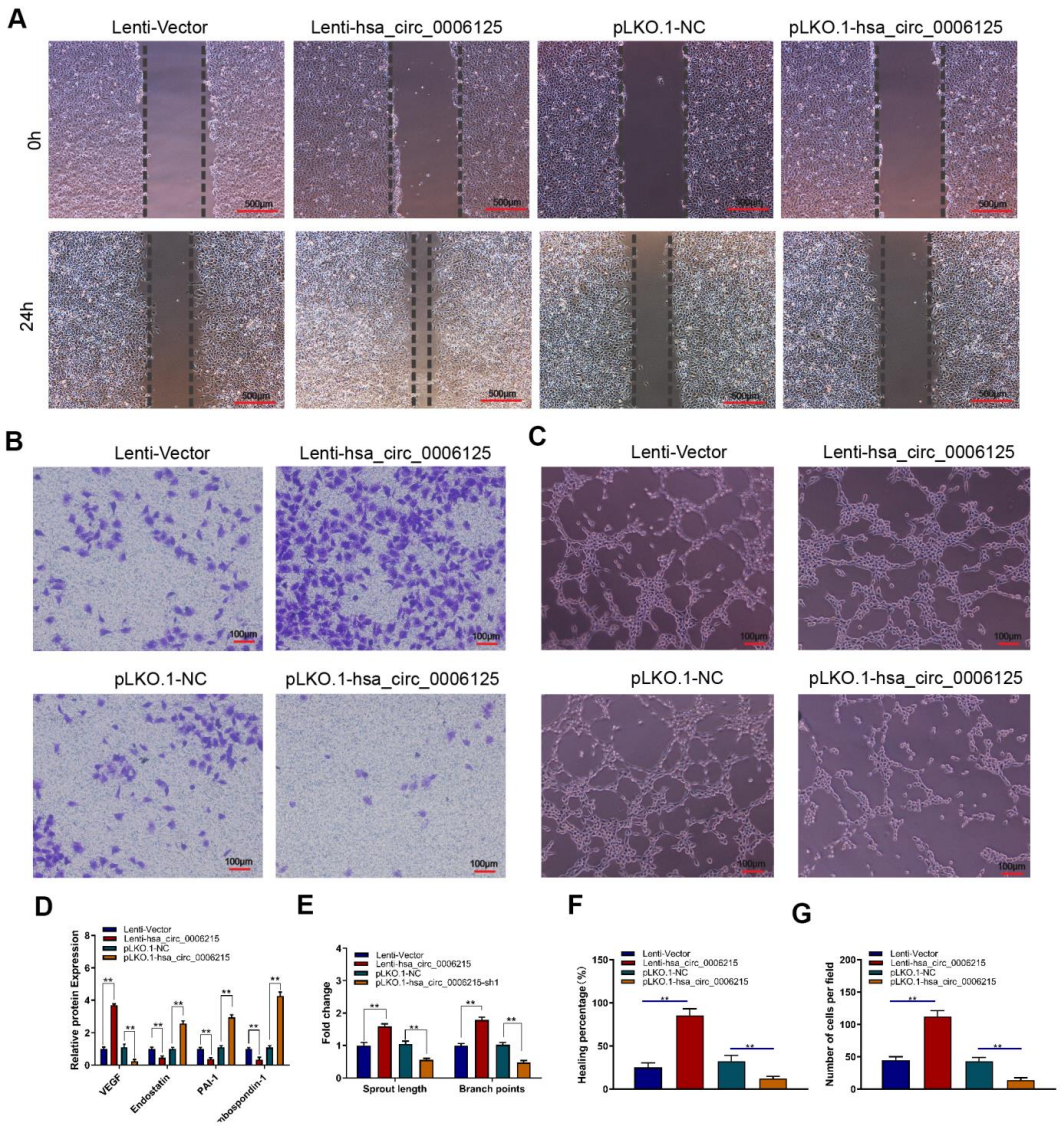
**CircRNA hsa_circ_0006215 promotes angiogenesis of HUVECs.** Effects of BMSCs culture supernatant migration after overexpression and knockdown of hsa_circ_0006215 on (**A**) HUVECs using scratch assays, (**B**) HUVECs invasion using Transwell™ assays, and (**C**) tube formation. (**D**) Effects of overexpressed and knocked down hsa_circ_0006215 on expression of angiogenesis-related factors in BMSCs culture supernatant detected by ELISA. Quantitation of (**E**) tube formation, (**F**) scratch assays and (**G**) transwell assays. Results are expressed as means ± SD of three independent experiments. *p < 0.05, **p < 0.01.

We assessed the effects of hsa_circ_0006215 on the coupling of new bone formation and angiogenesis. We overexpressed and knocked down hsa_circ_0006215 in BMSCs, removed the culture supernatant, and incubated them with HUVECs. The results of scratch (Figure 4A–4F) and Transwell™ (Figure 4B–4G) assays showed that culture supernatant of BMSCs overexpressing hsa_circ_0006215 significantly promoted HUVECs migration. The culture supernatant of BMSCs overexpressing hsa_circ_0006215 significantly promoted angiogenesis (Figure 4C–4E) and increased VEGF expression (Figure 4D) as shown by the results of enzyme-linked immunosorbent assay (ELISA). Western blot analysis showed that hsa_circ_0006215 did not affect angiopoietin-1 and FGF-2 protein expression (Supplementary Figure 1A). The culture supernatant of BMSCs overexpressing hsa_circ_0006215 and transfected with VEGF siRNA did not promote HUVECs migration (Supplementary Figure 1B).

### CircRNA hsa_circ_0006215 improves osteogenesis of BMSCs and bone regeneration *in vivo*

**Figure 5:**
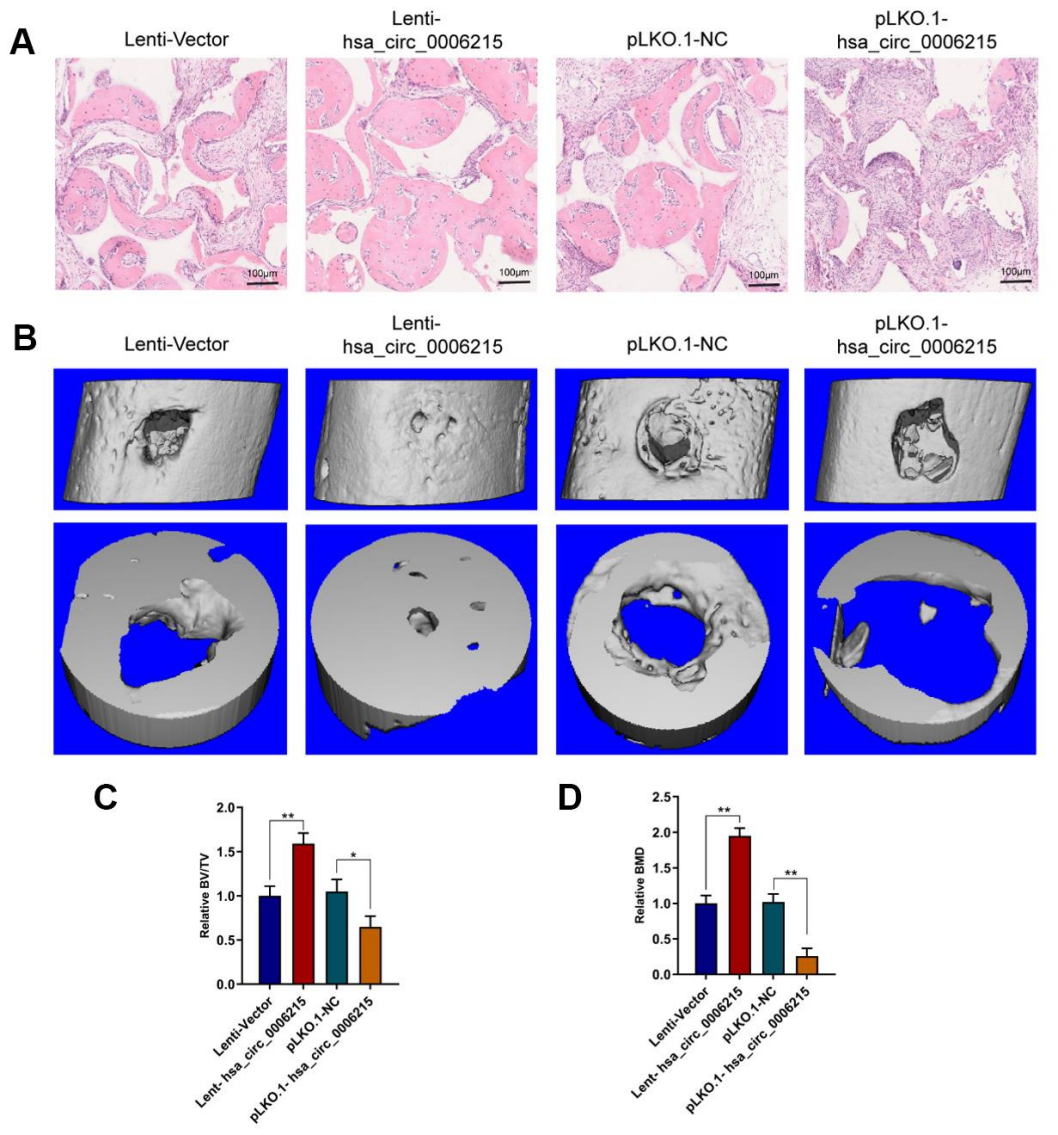
**CircRNA hsa_circ_0006215 promotes BMSCs osteogenesis and bone regeneration *in vivo*.** (**A**) Heterotopic osteogenic differentiation of BMSCs detected by HE staining. (**B**) New bone formation in cortical bone defect determined using MicroCT. MicroCT parameters (**C**) BV/TV and (**D**) BMD. All values are expressed as means ± SD of five samples. *p < 0.05, **p < 0.01. BMD, bone mineral density; BMSCs, bone marrow mesenchymal stem cells; BV/TV, bone volume per tissue volume; MicroCT, microcomputed tomography.

We determined the effects of hsa_circ_0006215 on the osteogenic differentiation of BMSCs using a heterotopic osteogenesis model *in vivo*. Overexpressed and knocked down hsa_circ_0006215 respectively promoted and inhibited the osteogenic differentiation of BMSCs *in vivo* (Figure 5A). Overexpressed and knocked down hsa_circ_0006215 respectively promoted and inhibited the repair of the single cortical bone defect in the model *in vivo* (Figure 5B–5D).

## DISCUSSION

We detected circRNA expression in BMSCs from individuals with or without senile OP using RNA-seq. The expression of some circRNAs significantly differed between the two groups, and expression of hsa_circ_0006215 in particular, was significantly changed during osteogenic differentiation of BMSCs. These results suggested that circRNA influences the development of senile OP by regulating BMSCs osteogenic differentiation. OP comprises primary, secondary, and idiopathic types; primary OP is classified as postmenopausal (Type I), which is closely related to the decline of ovarian function and decreased serum estrogen levels, while degenerative senile OP (Type II) is mainly caused by aging and affects the bone cortex and cancellous bone. Patients with OP are prone to vertebral, forearm, and femoral neck fractures. Although circRNAs related to postmenopausal OP have been investigated, those associated with senile OP remain unknown. Therefore, we investigated BMSCs from younger individuals without, and senile elderly patients using RNA-seq to identify circRNAs directly related to this pathology.

Through knockdown and overexpression of the expression of circRNA hsa_circ_0006215 in BMSCs, we found that this circRNA could promote osteogenic differentiation of BMSCs. We further studied the mechanism of regulation of this differentiation by hsa_circ_0006215. CircRNAs are ceRNA with microRNA response elements that function as microRNA sponges. The absence or presence of ceRNA regulates gene expression by affecting miRNA functional activity [16]. As a key regulator of gene expression at post-transcriptional level, microRNAs can bind to complementary gene sites according to the principle of base complementarity, and the changes in microRNAs can regulate the expression of genes and proteins. Complementary circRNAs can bind to their target microRNAs and inhibit their function. However, whether the sponge action is a universal function of circRNAs is still a topic of discussion. Osteogenic differentiation and vascularization of BMSCs are essential for bone health. During osteogenic differentiation, BMSCs promote angiogenesis by secreting vascular differentiation factors. We found that hsa_circ_0006215 regulated the expression of Runx2, which is an important osteogenic differentiation factor, and VEGF, which is an important pro-angiogenic factor, by regulating the function of miR-942-5p. Bone-specific H-type vessels are important in osteogenesis and their number decreases significantly with advancing age. Increasing the numbers of H-type vessels significantly improved BMD in aged mice. However, further studies are needed to decipher the effects of hsa_circ_0006215 on BMSC age. Hsa_circ_006215 may also affect the BMSCs osteogenic differentiation by regulating BMSCs aging. We did not examine microvessel density either in bone or surrounding the bone to show interactions between BMSCs and endothelial cells. This may have shown that VEGF-stimulated angiogenesis and osteoblast differentiation at bone-repair sites and strengthened our conclusions.

Despite these limitations, we verified that hsa_circ_0006215 promotes the osteogenic differentiation of BMSCs *in vivo*. With the development of high throughput sequencing technology and bioinformatics, attention has shifted to circRNAs. The mechanisms of circRNAs formation are varied. Different types of circRNAs perform different functions, such as the regulation of linear RNA transcription, as microRNAs and protein sponges, and in the translation of proteins. These complex functions enable circRNAs to participate in the occurrence and development of many diseases *via* various signaling pathways, and provide new insights into the pathogenesis of orthopedic diseases. Circular RNAs are structurally stable and they are abundant, tissue-, and disease-specific. They might therefore, serve as biomarkers for clinical diagnosis and as potential therapeutic targets. However, our *in vitro* results need to be verified *in vivo*. Therefore, further extensive investigations are necessary to fully understand the molecular mechanisms underlying the roles of circRNAs in the development of orthopedic diseases.

## MATERIALS AND METHODS

### Ethics statement

The study was conducted following the approval of the Ethics Committee of Zhoupu Hospital, Shanghai University of Medicine and Health Sciences (approval number: 2017-A-012-E011). The informed written consent was obtained from each participant. All animal care, handling, and surgical techniques followed protocols approved by the Animal Care and Use Committee of Zhoupu Hospital, Shanghai University of Medicine and Health Sciences (approval number: 2017-ZPYY-KW-17-314X).

### Isolation and culture of BMSCs

We collected BMSCs from four male patients with OP (average age, 86.2 ± 5.89 years) and from four male donors without OP (control; average age, 57.6 ± 9.56 years) and maintained them in α-modified essential medium (α-MEM; Sigma-Aldrich Corp., St. Louis, MO, USA) containing 10% fetal bovine serum (FBS) (Gibco) and 100 μg/mL penicillin-streptomycin sulfate (Sigma-Aldrich) at 37° C under a humidified 5% CO_2_ atmosphere [17]. For osteogenic differentiation, BMSCs at passage 3 (P3) were seeded at a density of 1 × 10^5^ /well in six-well plates and cultured in basal medium. Confluent cells were cultured in basal medium containing 10% fetal bovine serum, 100 U/mL penicillin, and 100 mg/mL streptomycin (DMEM) or with BM supplemented with 100 ng/mL BMP-2 (osteogenic medium) (#120-02; Inc., Rocky Hill, NJ, USA). The culture medium was changed every 3 days.

### RNA sequencing

The culture medium was removed, BMSCs (P1 generation) were lysed in TRIzol (1 mL per well), and RNA was extracted. The quality of RNA samples was assessed and they were used to construct a library. The RNA was sequenced and data were analyzed at Sangon Biotech (Shanghai) Co. Ltd., Shanghai, China.

### Lentivirus-mediated overexpression and knockdown

The pLKO.1-shRNA vectors targeting hsa_circ_0006215 and empty control vectors (scram-shRNA) (Sense sequence: AAGAAACTGCTAGGTCATAGA) were purchased from OriGene Technologies Inc. (Rockville, MD, USA) The lentiviruses were packaged using Lenti-vpak packaging kits (OriGene Technologies, Inc.) and transfected into BMSCs for 48 h using FuGENE^®^ HD Transfection Reagent (Roche Holdings AG, Basel, Switzerland). Viral supernatants were collected and used for infection. The hsa_circ_0006215 gene was ligated into the pCDH-circRNAs vector. Empty and pCDH-hsa_circ_0006215 constructs were transfected into viral packaging cells for 48 h. Supernatants were collected for BMSCs infection. Cells stably expressing green fluorescent protein (GFP) were collected using fluorescence-activated cell sorting.

### Quantitative real-time PCR (qRT-PCR)

Total RNA was extracted using TRIzol and reverse-transcribed using Prime-script RT reagent kits on a T100 thermal cycler (Bio-Rad Laboratories Inc., Hercules, CA, USA) as described by the manufacturer. The was used for real-time quantitative PCR performed using SYBR Green RT-qPCR Master Mix kits on a LightCycler 480 II (Roche Holdings AG), as described by the manufacturer. Relative gene expression was calculated using the 2^- ΔΔCT^ method. Amounts of mRNA and miRNA expression were respectively normalized with expression of glyceraldehyde 3-phosphate dehydrogenase (GAPDH) and U6. Supplementary Table 1 shows the sequences of all primers used in the present study.

### RNA pull-down assay

We co-transfected MS2bp-GFP and MS2bs-hsa_circ_0006215 or MS2bs-hsa_circ_0006215mut, or transfected a control (empty) plasmid into BMSCs using Lipofectamine 2000 or Renilla luciferase (MS2bs-Rluc). RNA precipitation was assayed 48 h later using EZ-Magna RIP RNA-Binding Protein Immunoprecipitation Kits (Millipore Sigma Co., Ltd., Burlington, MA, USA) and GFP antibody (Roche Holdings AG) as described by the manufacturer and the method described for MS2-RIP studies [18]. The RNA precipitate was retrieved and analyzed by qPCR. Cells were harvested by trypsinization, suspended in fresh phosphate buffered saline (PBS)-based nuclear isolation buffer (2 mL) and water (6 mL), and placed on ice for 20 min with frequent mixing. Nuclei were pelleted by centrifugation at 2,500 × *g* for 15 min, resuspended in fresh RIP buffer (1 mL), and divided into two 500-mL portions (mock and IP). Chromatin was mechanically sheared using a Dounce homogenizer with 15–20 strokes. The nuclear membrane and debris were pelleted by centrifugation at 13,000 rpm for 10 min. Ago2 antibody (10 μg) was added to the supernatant (10 mg) and the mixture was incubated for 2 h (to overnight) at 4° C with gentle rotation. Protein A/G beads (40 μL) were added to the mixture and incubated for 1 h at 4° C with gentle rotation. The beads were pelleted at 2,500 rpm for 30 s, the supernatant was removed, and the beads were resuspended in 500 mL of RIP buffer. This process was repeated three times and then, the beads were washed once with PBS. Coprecipitated RNA were isolated by resuspending the beads in TRIzol RNA extraction reagent.

### Western blotting

Cells were lysed on ice in RIPA protein lysate solution for 30 minutes. The cell debris was pelleted by centrifugation for 10 min at 10,000 rpm. Protein concentrations in the supernatant were determined using the bicinchoninic acid (BCA) method. Samples in sample buffer were heated for 10 min at 98° C. Samples containing 20 μg protein were resolved by electrophoresis on 10% sodium dodecyl sulfate-polyacrylamide (SDS-PAGE) gels using a vertical transfer system (#1658033 Bio-Rad Laboratories Inc.). The separated proteins were transferred onto polyvinylidene fluoride (PVDF) membranes. Nonspecific protein binding on the membranes was blocked with 5% BSA for 1 h and the membranes were incubated overnight at 4° C with the primary antibodies. The membranes were then washed with TBST for 1 h, incubated with HRP-labeled secondary antibodies, and finally washed with TBST. Bound proteins on the blots were visualized using an automatic chemiluminescence imaging analysis system (#5200; Tanon Science and Technology Co., Ltd., Shanghai, China). The primary antibodies specific to COL1A1 (#ab34710), RUNX2 (#ab192256), OCN (#ab13418), p-SMAD1 (#ab73211), t-SMAD1 (#ab33902), RUNX2 (#ab151302), VEGF (#ab52917), beta-catenin (#ab32572), Dicer (#ab227518), and GAPDH (#ab181602) were purchased from Abcam (Cambridge, UK).

### Osteogenic differentiation assays

After three rinses with PBS, the cell layer was fixed with 4% paraformaldehyde for 15 min at room temperature. The cells were then incubated with a buffer containing 0.1% naphthol AS-biphosphate and 2% fast violet B (both from Sigma-Aldrich Corp.). After incubation for 1 h at 37° C, the cell layer was washed with deionized water and ALP was assayed at 405 nm using p-nitrophenyl phosphate as substrate (pNPP; Sigma-Aldrich Corp.). Briefly, 50 mL of sample was incubated with 50 mL of pNPP (1 mg/mL) in 1 M diethanolamine buffer containing 0.5 mM MgCl_2_ (pH 9.8) at 37° C for 15 min. The reaction was stopped by adding 2 M NaOH to 200 μL of the reaction mixture. The total protein content was determined by the BCA method using a protein assay kit (PIERCE, Rockford, IL, USA). The activity of ALP was described as nmol of p-nitrophenol/min/mg protein and is presented as fold change relative to that in the control group. The cells were fixed in 70% ice-cold ethanol for 1 h, rinsed with double-distilled H_2_O, and stained with 40 mM Alizarin red S (pH 4.9; Sigma-Aldrich Corp.) for 15 min with gentle agitation. After five rinses with double-distilled H_2_O, the excess stain was removed from the cells using 10% (w/v) cetylpyridinium chloride (Sigma-Aldrich Corp.) for 1 h and mineralization was quantified by measuring absorbance at 570 nm.

### HUVECs scratch test

Human umbilical vein endothelial cells (HUVECs) (5 × 10^4^/well) were seeded in 6-well culture plates and cultured with DMEM containing 10% fetal bovine serum, 100 U/ml penicillin and 100 mg/mL streptomycin. When the cells grew to 100% confluent, the surface of the cells was scratched with a pipette tip, and the culture medium was aspirated. The cells were washed with PBS, incubated with BMSCs culture supernatants for 24 h, and photographed using a microscope (Leica, Wetzlar, Germany).

### Transwell migration analysis of HUVECs

We seeded HUVECs (1 × 10^4^ /well) suspended in serum-free medium into the upper chamber of transwells and added FBS (1%) to the lower chamber. The culture scaffold and the cells in the upper chamber were removed after 24 h and the cells were stained with 0.5% crystal violet and counted.

### Tube formation analysis in HUVECs

Serum-free medium containing BD Matrigel™ matrix (100 μL) was added to 96-well plates, and incubated for 45 min at 37° C. Thereafter, HUVECs (1 × 10^4^/well) that had been cultured in BMSCs supernatant were seeded in 96-well plates containing Matrigel™ then photographed 4 h later.

### Fluorescence *in situ* hybridization

Cells on slides were fixed with 4% paraformaldehyde at 4° C for ~3 h, immersed in protease K buffer at 37° C for 20 min, washed with sterilized water for 1 min three times, dehydrated with a graded series of 50%, 80%, and 100% ethanol, and dried in air. Thereafter, 9 μL of hybridization buffer and 1 μL probe was added to the slides and the slides were washed with heated flushing solution, sealed with a fluorescence quenching agent, and fluorescence emission was measured.

### Ectopic bone formation

We assessed ectopic bone formation as described earlier [19]. We resuspended BMSCs at a density of 5 × 10^6^ cells/mL. A beta tricalcium phosphate scaffold (β-TCP; Φ < 1.0 mm; Shanghai Bio-lu Biomaterials Co., Ltd., Shanghai, China) (0.5 mL), was incubated with BMSCs suspension (45 mg/well) in 48-well plates for 12 h. Thereafter, β-TCP coated with BMSCs was subcutaneously transplanted into the backs of 8-week-old male nude mice (n = 6). The mice were sacrificed by cervical dislocation 2 months later and the transplant was removed. New bone formation was detected as EDTA decalcification and hematoxylin and eosin (HE) staining.

### Mouse models of femoral monocortical defect

We created mouse models of femoral monocortical defects as described earlier [20]. BMSCs derived from patients with OP were collected at P3 and 5 × 10^4^ cells suspended in medium containing Matrigel™ (10 μL) were transplanted into the osseous hole (0.8 mm diameter) in the right femurs of nude mice. Two weeks later, the femurs were isolated, fixed overnight in 4% neutral buffered formalin, and stored in 70% ethanol until analyzed using a μCT35 system (Scanco Holding AG, Brüttisellen, Switzerland) with a spatial resolution of 5 μm. Sagittal sections of injured femur were analyzed by 3D histomorphometry.

### Statistical analysis

Data were statistically analyzed using SPSS 23.0 (IBM Corporation, Armonk, NY, USA). Experimental results are shown as means ± SD (of tissue preparations, cells, or experimental replicates). Multiple groups were compared using Student-Newman-Keuls multiple comparison tests. If equal variances were not assumed, between-group differences were compared using Dunnett T3 tests. Results with *p* < 0.05 were considered statistically significant.

## Supplementary Material

Supplementary Figure 1

Supplementary Table 1
