# Supplementary material for: CircRNA hsa_circ_0006215 promotes osteogenic differentiation of BMSCs and enhances osteogenesis–angiogenesis coupling by competitively binding to miR-942-5p and regulating RUNX2 and VEGF
Source: Aging (Albany NY). 2021 Apr 4;13(7):10275–88. doi: 10.18632/aging.202791 (PMC8064180; doi:10.18632/aging.202791)
Supplement: Supplementary Figure 1 [file aging-13-202791-s001.pdf]

SUPPLEMENTARY FIGURE

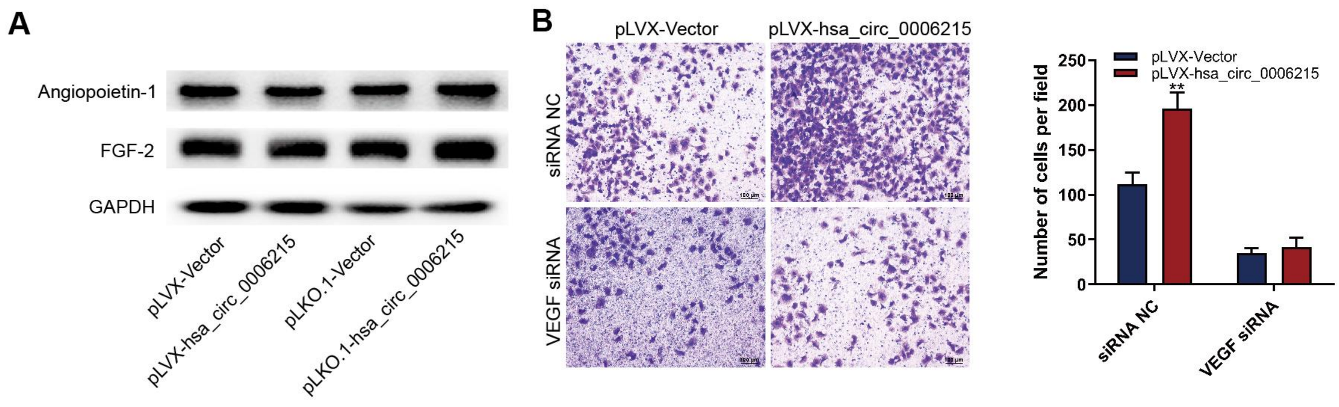

Supplementary Figure 1. CircRNA hsa_circ_0006215 enhances osteogenesis–angiogenesis coupling by promoting VEGF expression. (**A**) Western blots show angiopoietin-1 and FGF-2 expression. (**B**) Transwell assays of HUVECs migration with VEGF knockdown in BMSCs. BMSCs, bone marrow mesenchymal stem cells; HUVECs, human umbilical vein endothelial cells; VEGF, vascular endothelial growth factor.
