# Supplementary material for: CircRNA hsa_circ_0006215 promotes osteogenic differentiation of BMSCs and enhances osteogenesis–angiogenesis coupling by competitively binding to miR-942-5p and regulating RUNX2 and VEGF
Source: Aging (Albany NY). 2021 Apr 4;13(7):10275–88. doi: 10.18632/aging.202791 (PMC8064180; doi:10.18632/aging.202791)
Supplement: Supplementary Table 1 [file aging-13-202791-s002.pdf]

SUPPLEMENTARY TABLE

Supplementary Table 1. Primer sequences for QRT-PCR.

Primer name	Forward primer sequence	Reverse primer sequence
hsa_circ_0006215	CCCCATGATCTCTTCACGGA	GTGGTGATGTTTGTGTCCGC
hsa_circ_0016033	CTCCTCCATCAGCTTGTGACC	CACAACAGTAGCCGGTCTTCA
hsa_circ_0062045	GGCCCCCTTTCTATGTTCATGT	CATCTGGCTGTGGCTGTACT
hsa_circ_0069139	AGAGACGGGATCGTCCACAG	TGCCTTTTTCTCCCTGACAGTT
hsa_circ_0082036	TCCAAGGAAGTCATGTGCCAG	GCCCAGATCACCTTTTGATGT
hsa_circ_0050199	GCCATTGTCTCCGAAGCGTA	CCACAGGATGAACAGGGGAG
hsa_circ_0058641	TCACACTTCCTGTCATTGGCT	CTATGAGTGCCCCTTGCCTC
hsa_circ_0040796	TGGCTAACCTGTTACACTGGG	CTGTGGGTGGATCATGGAGAG
hsa_circ_0030026	CAGGTCTGGTACCATTAGCGA	ATTGCCTTCAACTGGGGCTC
hsa_circ_0047940	TGTGGTGATCCAGTCATGCG	TCCATTGCAAAGTGAAGACACG
hsa_circ_0035557	CAGGTCTGGTACCATTAGCGA	GCTCGGCCCAGGAAAAATATC
hsa_circ_0048410	TTTTAGTGTGGGCAGCTGGT	CTGCTCTGGCCCTTCCAC
hsa_circ_0073824	AGATGCAGCTCGTGGATACG	AACAGATAGTTGGAGAAAGGACC
hsa_circ_0059514	GGTCCTCGAGAGAAGATGCAG	GCAGTGTGGTGCTTCCAGTT
hsa_circ_0062856	TACGGTACTTCCTGCCTCCA	TTCGTGAGTGGTTCAGAGGA
hsa_circ_0062847	TTGGTTTGACTTACACGGGACA	AGCAAGGGCACCAAACAAGA
hsa_circ_0013330	ATCCTCCGACCCTCCTACAC	GGTACGTGGCTGTCTCTGAC
hsa_circ_0061541	AGTTGGCCAATACTGGTGCC	AGGTTCTTCTTCCTCTTCATCCG
hsa_circ_0019789	ACAAAATTGGAAAAAGATTGGCA	ACACAATTCACAAATTCTTTAC
hsa_circ_0049502	AGGCTGGTCTCGAACTCCTGAC	AGGCTGGTCTCGAACTCCTGAC
hsa_circ_0048536	AGGTGGTGAAACAATCTTGATGA	TTCCCACAAGGCACATCCTC
GAPDH	ATGTTGCAACCGGGAAGGAA	AGGAAAAGCATCACCCGGAG
VEGF	GTCCTGGAGCGTGTACGTTG	CTTCCGGGCTCGGTGATTTA
RUNX2	CGCCTCACAAACAACCACAG	TCACTGTGCTGAAGAGGCTG
